# Topical application of endothelin receptor a antagonist attenuates imiquimod-induced psoriasiform skin inflammation

**DOI:** 10.1038/s41598-020-66490-z

**Published:** 2020-06-11

**Authors:** Takeshi Nakahara, Makiko Kido-Nakahara, Dugarmaa Ulzii, Sho Miake, Kei Fujishima, Sawako Sakai, Takahito Chiba, Gaku Tsuji, Masutaka Furue

**Affiliations:** 1 Division of Skin Surface Sensing, Graduate School of Medical Sciences, Kyushu University, Fukuoka, Japan; 2 Department of Dermatology, Graduate School of Medical Sciences, Kyushu University, Fukuoka, Japan; 3 Department of Dermatology and Plastic Surgery, Akita University Graduate School of Medicine, Akita, Japan; 4 Research and Clinical Center for Yusho and Dioxin, Kyushu University, Fukuoka, Japan

**Keywords:** Inflammation, Skin diseases

## Abstract

Endothelin-1 (ET-1) is well known as the most potent vasoconstrictor, and can evoke histamine-independent pruritus. Recently, its involvement in cutaneous inflammation has begun to draw attention. The upregulation of ET-1 expression in the epidermis of human psoriasis patients has been reported. It was also demonstrated that ET-1 can stimulate dendritic cells to induce Th17/1 immune responses. However, the role of the interaction between ET-1 and ET-1 receptors in the pathogenesis of psoriasis remains elusive. Here, we investigated the effects of ET-1 receptor antagonist on imiquimod (IMQ) -induced psoriasiform dermatitis in mouse. Psoriasis-related cytokines such as IL-17A and TNF-α induced ET-1 expression in human keratinocytes. Topical application of selective endothelin A receptor (ETAR) antagonist ambrisentan significantly attenuated the development of IMQ-induced psoriasiform dermatitis and also significantly inhibited the histological inflammation and cytokine expression (TNF-α, IL-12p40, IL-12 p19, and IL-17) in the lesional skin of the mouse model. Furthermore, topical application of ambrisentan suppressed phenotypic and functional activation of dendritic cells in lymph nodes. Our findings indicate that the ET-1 and ETAR axis plays an important role in the pathogenesis of psoriasis and is a potential therapeutic target for treating psoriasis.

## Introduction

Psoriasis is a chronic, T-cell-mediated inflammatory skin disease associated with both genetic and environmental factors^1^. In the process of developing of psoriasis, keratinocytes and immune cells interact with each other through the secretion of cytokines and chemokines^2–4^. This cellular interaction, especially through the interleukin (IL)-23/Th17 cytokine axis, ultimately leads to psoriatic inflammation and skin lesions such as scaly erythema and scaly plaques. That is, IL-23 secreted by dendritic cells, stimulates and maintains Th17 cells. These activated Th17 cells secrete IL-17A and IL-22, which finally induce proliferation of keratinocytes^5^. The topical application of imiquimod (IMQ), a toll-like receptor (TLR)7 ligand and a potent immune activator, induces skin inflammation in mice with features similar to psoriasis via the IL-17/23 axis^6^. In this model, mice are topically applied with IMQ cream for 5 or 6 days. This treatment results in rapid inflammation in the skin, which resembles the clinical and histopathological findings of human psoriasis^7^. In addition, this model closely reflects the cytokine profile of human psoriasis, especially that of the IL-23/Th17axis^7^. Therefore, this model could be one approach to analyse the immunological aspects of human psoriasis.

Endothelin-1 (ET-1), a 21-amino-acid peptide, is expressed by various cells such as endothelial cells, neurons, immune cells, and keratinocytes^8–10^. It is known that ET-1 is one of the most potent vasoconstrictor compounds and is upregulated in response to hypoxia, stress, and inflammatory cytokines^11–14^. ET-1 exerts its biological action through the activation of two G-protein-coupled receptors: endothelin A receptor (ETAR) and endothelin B receptor (ETBR). There are two types of ET receptor antagonist: one is the ETAR-selective antagonist ambrisentan and the other is the dual ETAR and ETBR antagonist bosentan. ETAR generally plays critical roles in the signalling pathway of ET-1, while ETBR occasionally counteracts ETAR activity^15^. In addition to its vasoconstrictive properties, some of the important functions of ET-1 include regulating cell signalling and inflammatory processes^16–18^, and increased ET-1 expression is found in many diseases^19–23^. Besides its systemic functions, ET-1 exerts important biological functions in the skin, including in keratinocyte proliferation, leukocyte migration, and angiogenesis, which are also characteristic features of psoriasis^10,24^. Furthermore, it was recently reported that ET-1-stimurated bone-marrow-derived dendritic cells (BMDCs) prime T cells to produce Th1 and Th17 cytokines^18^. These findings indicate that ET-1 is likely to be involved in the inflammatory processes of psoriasis. However, the role of ETAR and ETBR in the pathogenesis of psoriasis remains elusive. In this study, we analysed whether ambrisentan or bosentan modulates the IMQ-induced murine model of psoriasiform dermatitis.

## Results

### ET-1 expression increases in IMQ-induced psoriasiform dermatitis in mice

**Figure 1:**
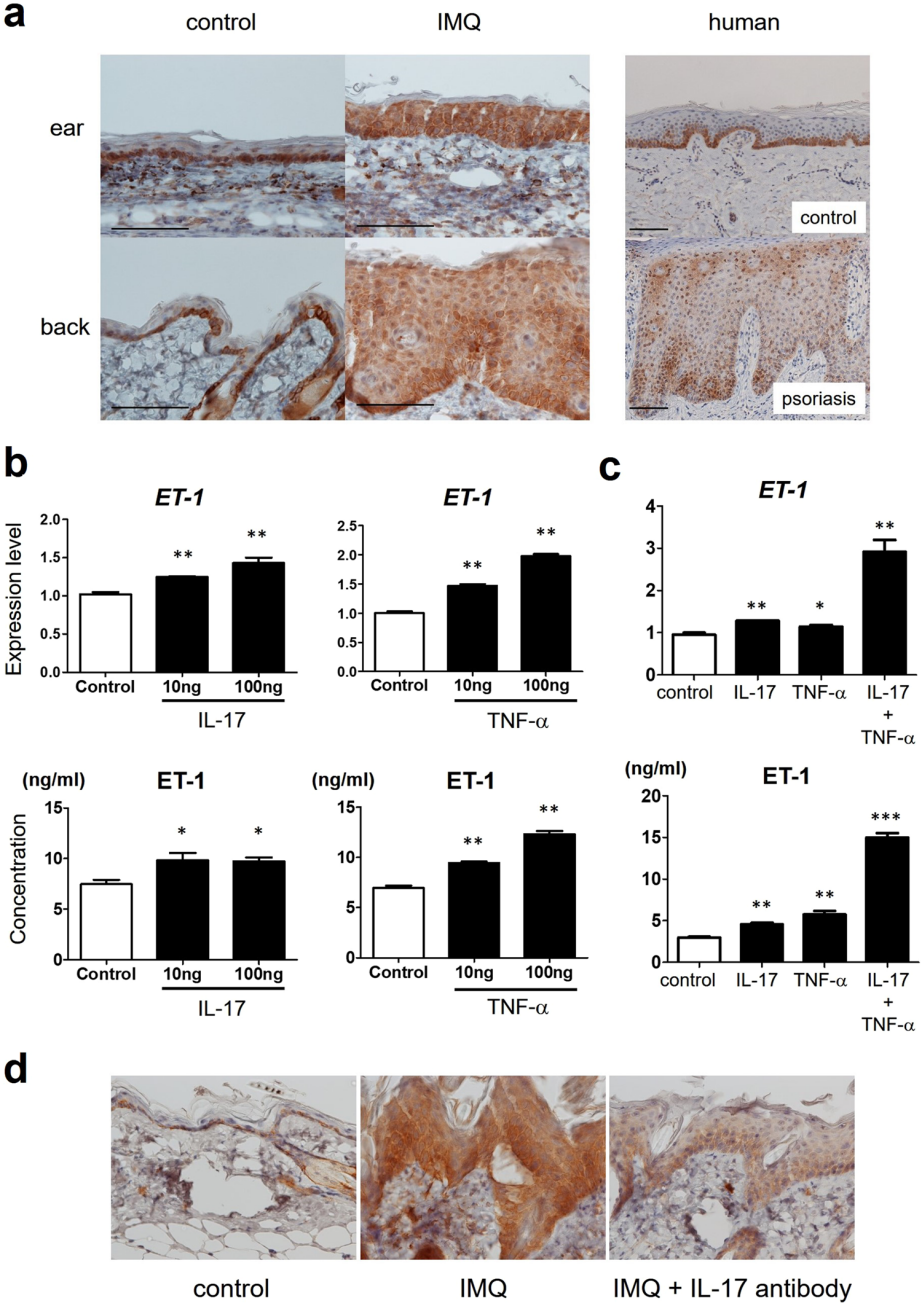
The expression of ET-1 in mouse and human psoriasis. (**a**) Immunohistochemical staining for ET-1 in normal skin and psoriasis. Expression of ET-1 was preferentially confined to basal keratinocytes in control mouse or normal human skin (n = 5). In IMQ-induced murine psoriasiform dermatitis (n = 5) or human psoriasis (n = 5), ET-1 expression was detected widely in the whole epidermis. Scale bar: 50 μm. (**b**) NHEKs were cultured with or without IL‐17 or TNF-α for 24 h. (**c**) NHEKs were cultured with or without IL‐17, TNF-α, or both for 24 h. Expression levels of mRNA of ET-1 in NHEKs were determined using quantitative PCR. Concentrations of released ET-1 were also measured in cell-free supernatants by ELISA. Data are shown as mean ± SEM. Results are representative of similar results obtained in three independent experiments. *P < 0.05, **P < 0.01 versus the control group (without IL-17 or TNF-α treatment). (**d**) ET-1 expression in psoriatic epidermis after local application of IL-17 neutralizing antibody. Mice were applied topical IMQ cream daily for five days. At day 1 and day 4, mice were administered IL-17 neutralizing antibody (150 μg/40 μl). Samples from the back at day 6 from control mice (n = 2), IMQ-treated mice (n = 2), and IMQ-treated mice with IL-17 neutralizing antibody (n = 2) were stained for ET-1.

In line with a previous study^18^, ET-1 expression was increased in the epidermis of human psoriasis patients compared with that in normal control epidermis (Fig. 1a). In addition, the expression levels of ET-1 were significantly increased in the epidermis of IMQ-induced psoriasiform dermatitis in mouse (Fig. 1a). ET-1 was expressed both in the cytoplasm and in the nucleus. Although ET-1 was expressed strongly in the basal cell layer of control human and murine skin, the expression spread throughout the epidermis in human and murine psoriatic skin. It is well known that IL-17A and TNF-α are highly expressed locally in psoriatic skin^3^. To examine the regulation of ET-1 expression in the epidermis in psoriasis, we investigated the expression of ET-1 in human keratinocytes after stimulation with psoriasis-related cytokines such as IL-17A and TNF-α. Each of the cytokines increased the mRNA and protein levels of ET-1 in human keratinocytes (Fig. 1b). In addition, the two cytokines synergistically increased the mRNA and protein levels of ET-1 in combination (Fig. 1c). To evaluate the regulation of ET-1 expression by IL-17 in psoriatic epidermis *in vivo*, we also investigated the inhibitory effect of IL-17 neutralizing antibody on IMQ-induced increase of ET-1 expression. Local application of IL-17 neutralizing antibody suppressed IMQ-induced increase of ET-1 expression in psoriatic epidermis *in vivo* (Fig. 1d).

### Topical application of selective ETAR antagonist ambrisentan prevents the development of IMQ-induced psoriasiform dermatitis in mice

High expression of ET-1 may be involved in inflammatory processes associated with psoriasis. To investigate whether there is a beneficial effect in psoriasis, the selective ETAR antagonist ambrisentan was topically applied to the mouse model. Ambrisentan was applied daily for 4 days, after which the mice were challenged topically on the ears and back skin with IMQ. Clinical scores for disease severity were calculated daily using a scoring system based on three clinical items (erythema, scales, and thickness).

**Figure 2:**
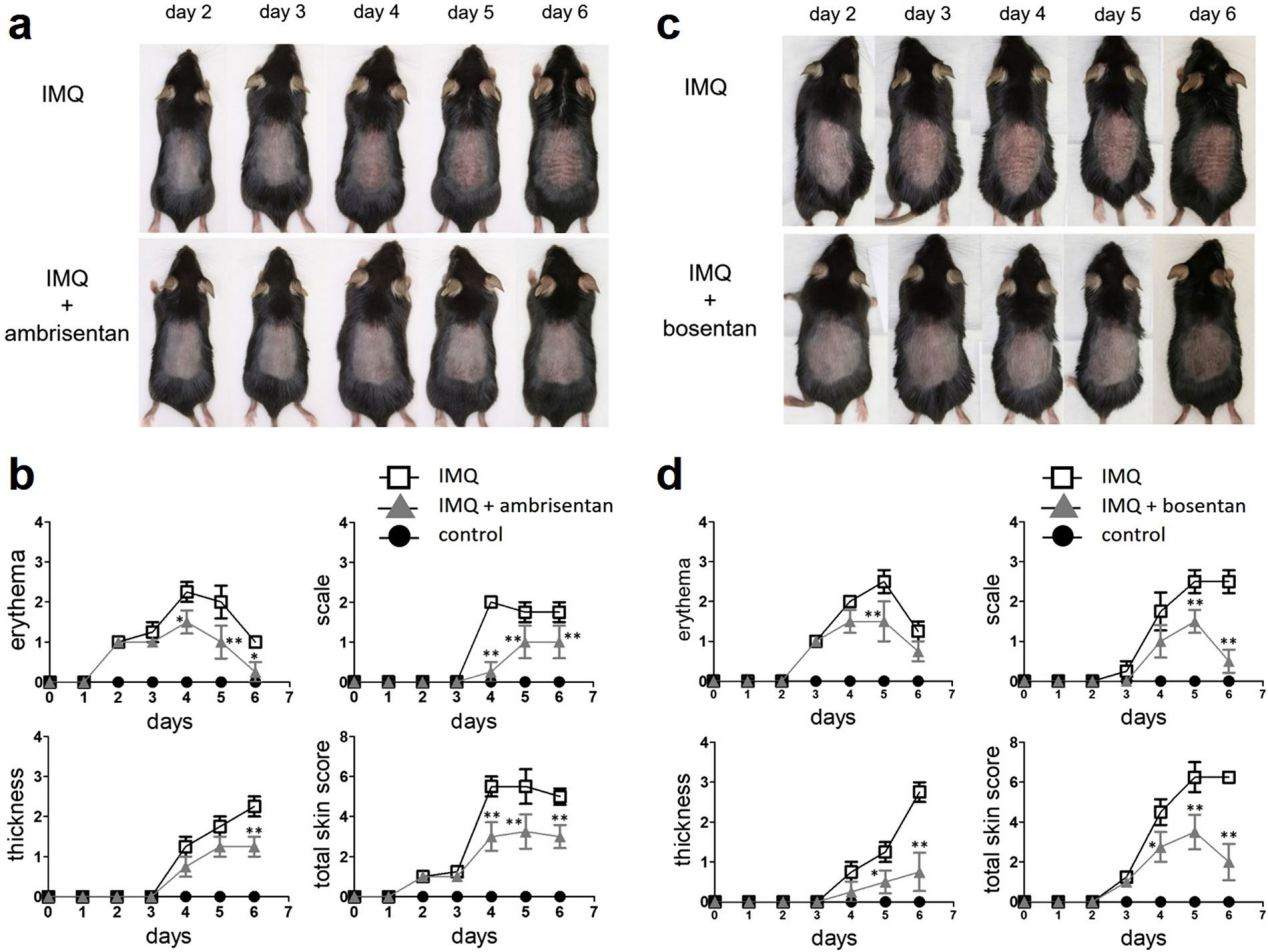
The effects of topical application of ambrisentan or bosentan on clinical findings of IMQ-induced psoriasiform dermatitis. Shaved back skin and ears of B6 mice were topically treated with IMQ or control vehicle for 6 consecutive days. Topical ambrisentan or bosentan was administered from 4 days before IMQ application until the end of the study. (**a,c**) Pictures of mice were taken and the phenotypic symptoms of mouse skin were observed from day 0 to day 6. (**b,d**) Clinical scores for disease severity were calculated daily using a scoring system based on the clinical Psoriasis Area and Severity Index. Erythema, scales, and thickness were scored independently on a scale from 0 to 4: 0, none; 1, slight; 2, moderate; 3, marked; and 4, very marked. The cumulative score (erythema, scales, and thickness) served as a measure of the severity of inflammation (scale 0–12). Results are representative of similar results obtained in three independent experiments. Data are presented as mean ± SEM (n = 5 for each group). *P < 0.05, **P < 0.01 versus IMQ-treated group.

Significant differences in clinical skin score were observed between IMQ mice and IMQ mice treated with ambrisentan from day 4 to 6 (Fig. 2a). Ambrisentan improved erythema from day 4 to 6, scales from day 4 to 6, and thickness at day 6 (Fig. 2b). Topical application of the dual ETAR and ETBR antagonist bosentan also alleviated the clinical changes of IMQ-induced psoriasiform dermatitis, but only at later time points (Fig. 2c,d). Specifically, it improved erythema at day 5, and scales and thickness from day 5 to 6 (Fig. 2c,d). On the other hand, the selective ETBR antagonist BQ-788 did not show any effects of improving the clinical changes of IMQ-induced psoriasiform dermatitis (Supplemental Fig. S1).

### Topical application of ambrisentan alleviates the histological changes of IMQ-induced psoriasiform dermatitis in mice

**Figure 3:**
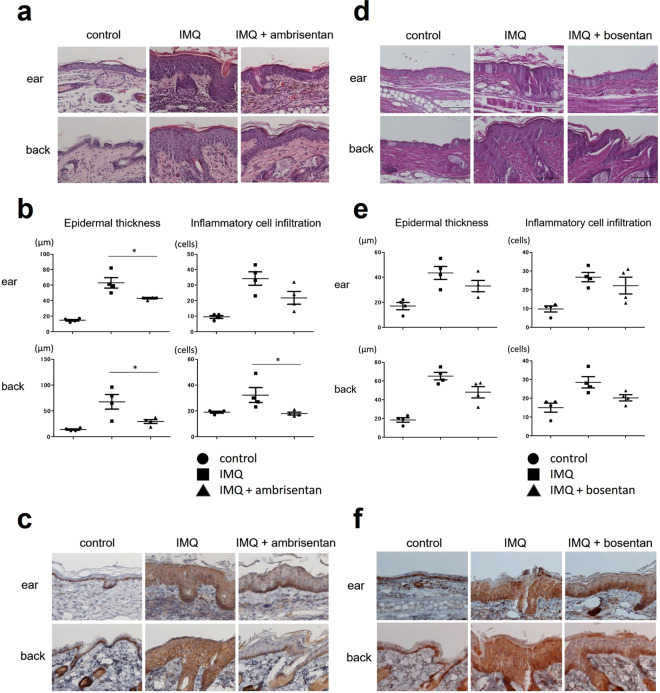
Histological presentation of control skin, IMQ-induced psoriasiform dermatitis, and IMQ-induced psoriasiform dermatitis treated with ambrisentan or bosentan. (**a,d**) H&E staining of skin tissue harvested at day 6 (×400). Samples from the ear and back of control mice, IMQ-treated mice, and IMQ+ ambrisentan or bosentan-treated mice at day 6 (after treatment for five consecutive days with IMQ) were stained using H&E. (**b,e**) Epidermal thickness and inflammatory cell infiltration of control skin, IMQ-induced psoriasiform dermatitis, and IMQ-induced murine psoriasiform dermatitis treated with ambrisentan or bosentan. The epidermal thickness and number of dermal inflammatory cells were determined per high-power field from four mice per group. Data are presented as mean ± SEM. *P < 0.05, versus IMQ-treated group. (**c,f**) Immunohistochemical staining for ET-1 of samples from the ear and back of control mice, IMQ-treated mice, and IMQ+ ambrisentan or bosentan-treated mice (×400). Results are representative of similar results obtained in three independent experiments.

Histopathologically, psoriasis is mainly characterized by epidermal hyperplasia and inflammatory cell infiltration^2^. Consistent with the clinical findings, histological analyses of IMQ-induced psoriasiform dermatitis at day 6 showed severe epidermal hyperplasia and intense inflammatory cell infiltration compared with the case in control mice (Fig. 3a). Ambrisentan treatment significantly improved the histological changes induced by IMQ treatment, attenuating the epidermal hyperplasia and inflammatory cell infiltration in both the ear and the back skin (Fig. 3a,b). In addition, ambrisentan treatment decreased the expression levels of ET-1 in the epidermis of IMQ-induced psoriasiform dermatitis (Fig. 3c). On the other hands, bosentan treatment partially, and not significantly, alleviated the histological features of IMQ-induced psoriasiform dermatitis (Fig. 3d–f).

### Topical application of ambrisentan significantly inhibits the expression of psoriasis-related cytokines in the lesional skin of IMQ-induced psoriasiform dermatitis in mice

**Figure 4:**
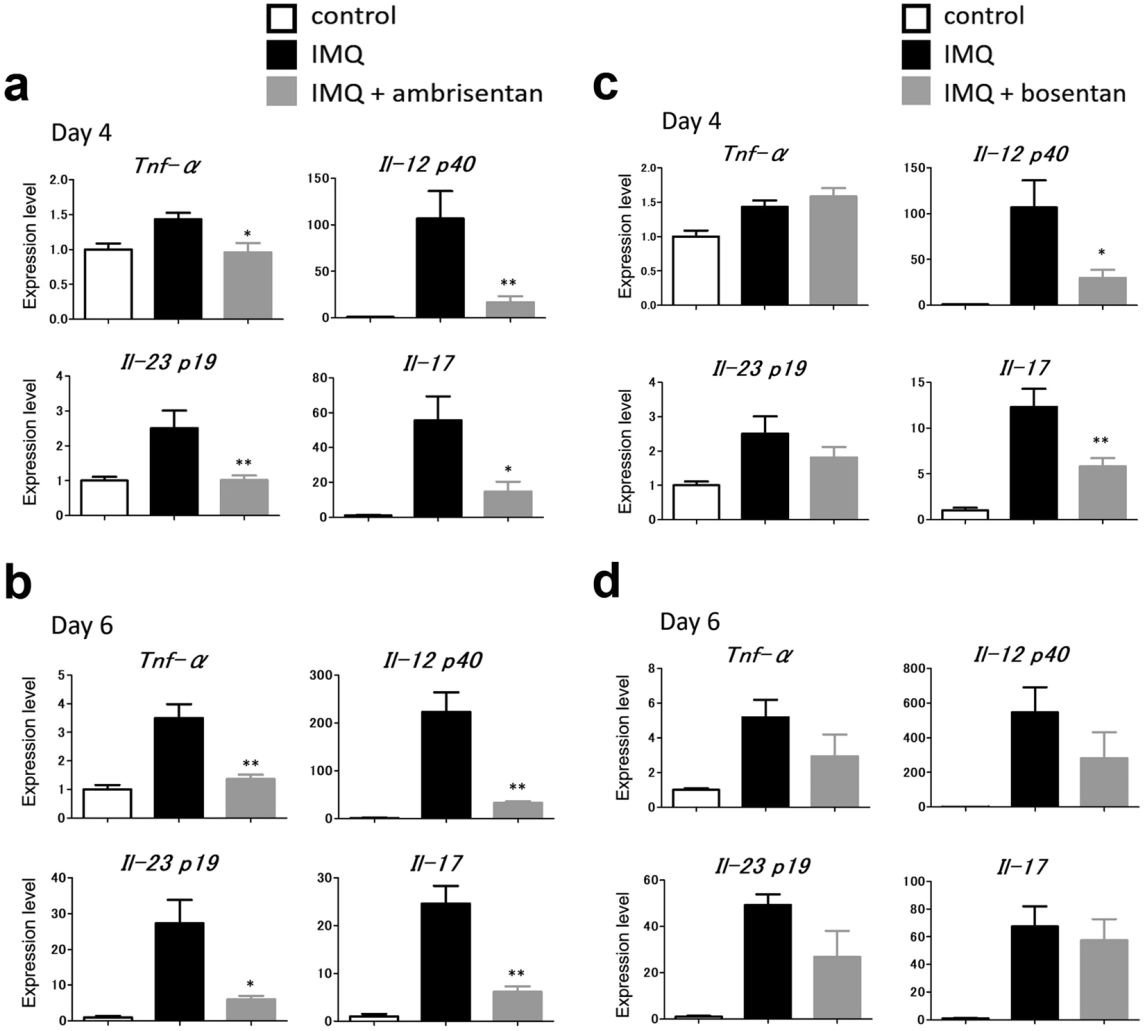
The expression of psoriasis-related cytokines in the lesional skin of control skin, IMQ-induced psoriasiform dermatitis, and IMQ-induced psoriasiform dermatitis treated with ambrisentan. RNA was obtained from the ear skin of control mice, IMQ-treated mice, and IMQ+ ambrisentan or bosentan-treated mice at day 4 (**a,c**) or day 6 (**b,d**). mRNA levels of TNF-α, IL-12 p40, IL-23 p19, and IL-17 were determined by quantitative PCR. Results are representative of similar results obtained in three independent experiments. Data are presented as mean ± SEM (n = 4 for each group). *P < 0.05, **P < 0.01 versus IMQ-treated group.

We next examined the mRNA levels of psoriasis-related cytokines in the IMQ-induced psoriasiform skin lesions with or without ambrisentan treatment. Skin samples were taken from mouse ear skin 4 or 6 days after IMQ application. mRNA levels of TNF-α, IL-12p40, IL-23p19, and IL-17 were determined by qRT-PCR. IMQ-treated skin demonstrated increased levels of mRNA encoded by the genes mentioned above, and ambrisentan treatment significantly decreased the mRNA levels of TNF-α, IL-12 p40, IL-23 p19, and IL-17 at day 4 and day 6, which should reflect the anti-inflammatory roles of ambrisentan (Fig. 4a,b). By contrast, the effects of bosentan on cytokine expression were very limited. Bosentan treatment only decreased the mRNA levels of IL-12 p40 and IL-17 at day 4 (Fig. 4c,d).

### Topical application of ambrisentan significantly inhibits the phenotypic and functional activation of DCs in draining lymph nodes

**Figure 5:**
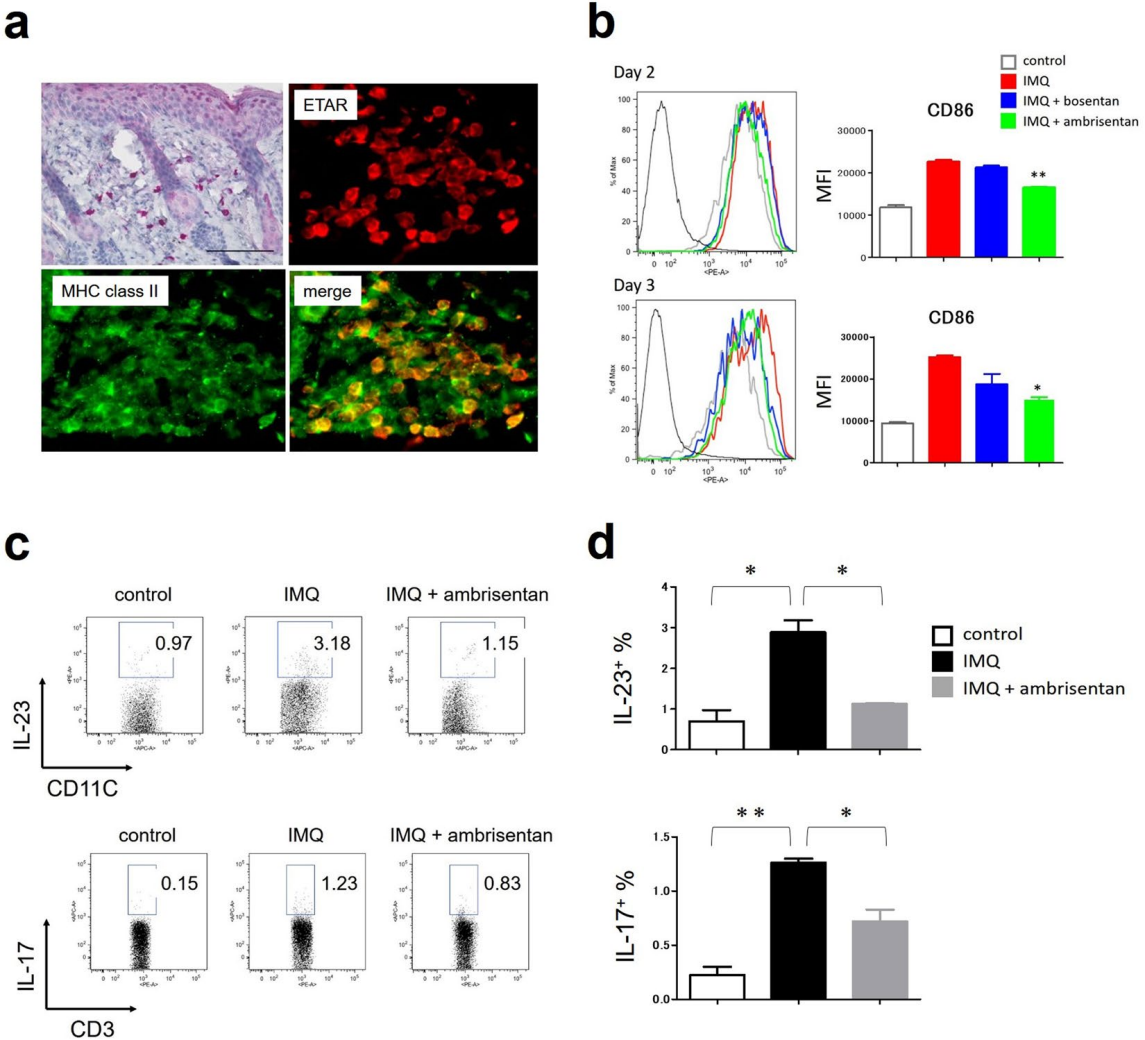
The effect of topical application of ambrisentan on the phenotypic and functional activation of sDCs in draining lymph nodes. Inguinal LNs of control mice, IMQ-treated mice, and IMQ+ ambrisentan-treated mice were harvested at day 2 or day 3. (**a**) Immunohistochemical staining for ETAR of samples from the back of IMQ-treated mice (left, upper panel). Immunofluorescence imaging of ETAR-positive and MHC class II-positive cells in the back of IMQ-treated mice. Results are representative of at least two separate experiments. (**b**) The expression of CD86 on skin-derived DCs in LNs was analysed by flow cytometry. Results are representative of similar results obtained in two independent experiments. Data are presented as mean ± SEM (n = 3 for each group). (**c,d**) The percentage of IL-23-producing sDCs or IL-17-producing T cells was examined by flow cytometry. Representative flow plots (**c**) and representative data from two independent experiments (**d**) are shown. Data are presented as mean ± SEM (n = 2 for each group). *P < 0.05, **P < 0.01 versus IMQ-treated group.

To identify the targets of ambrisentan, we examined the expression of ETAR in the skin of control mice or IMQ-treated mice. Immunohistochemical staining revealed that both epidermis and infiltrated immune cells express ETAR (Fig. 5a). Among infiltrated immune cells, most of the ETAR-positive cells coexpressed MHC class II (Fig. 5a). Therefore, considering that ambrisentan could act on both epidermis and DCs, we first examined the self-regulatory effect of ET-1 on the proliferation of keratinocytes, and the effects of ambrisentan on epidermal proliferation. As previously reported, ET-1 prompted the proliferation of NHEKs *in vitro*. However, this effect was only slight and not significant. Ambrisentan showed a tendency to suppress the promotion of NHEK proliferation by ET-1 (Supplemental Fig. S2). Next, we examined the effects of ambrisentan on the phenotypic and functional activation of DCs in draining lymph nodes. T-cell activation by DCs is an important factor in the development of psoriasis, and the maturation status of DCs determines the T-cell differentiation. In secondary lymphoid tissues, migratory DCs and resident DCs (rDCs) are discriminated by their relative expression levels of CD11c and MHC class II. In LNs, most of the migratory DCs are skin-derived DCs (sDCs) and have a CD11c^int^ MHC class II^high^ phenotype, while rDCs have a CD11c^high^ class II^int^ phenotype (Supplemental Fig. S3)^25^. We harvested inguinal LNs at day 2 and day 3, and analysed the phenotypic and functional activation status of sDCs by flow cytometry. As expected, IMQ treatment induced higher expression of CD86 on sDCs in LNs (Fig. 5b). Pretreatment of ambrisentan significantly decreased the expression of CD86 on sDCs in LNs at day 2 or day 3 (Fig. 5b). In contrast, bosentan only slightly, but not significantly, decreased the expression of CD86 on sDCs in LNs (Fig. 5b). Pretreatment of ambrisentan also decreased the expression of CD80 on sDCs in LNs at day 2 or day 3 (Fig. S4). As a functional change of DCs, IL-23 secretion was evaluated. As well as phenotypic maturation, IMQ also increased the percentage of IL-23-producing sDCs in LNs, and ambrisentan suppressed this proportion (Fig. 5c). We also evaluated the percentage of IL-17-producing T cells in LNs. The effect of ambrisentan on the percentage of IL-17-producing T cells was similar to that on IL-23-producing sDCs. Ambrisentan suppressed the proportion of IL-17-producing T cells increased by IMQ (Fig. 5d).

## Discussion

Regarding the relationship between ET-1 and skin diseases, it is well known that ET-1 causes itch^26–29^, which is one of the most troublesome symptoms of skin disease. However, in recent years, an association of ET-1 with cutaneous inflammation has been reported. For example, it was reported that ET-1 amplified epidermal inflammation via cytokine production in atopic dermatitis (AD)^30^. ET-1 augmented IL-25 expression in keratinocytes, and IL-25 reciprocally increased ET-1 expression^30^. This vicious circle of mutual positive feedback between ET-1 and IL-25 may be part of the pathogenesis of AD. It was also reported that ET-1 was expressed in the epidermis of psoriasis^18^. ET-1 directly induced phenotypic maturation of BMDCs, and ET-1-activated BMDCs primed T cells to produce Th17/1 cytokines, which have central roles in the development of psoriasis^18^. However, the roles of ET-1 and its specific receptors ETAR and ETBR in IMQ-induced psoriasiform dermatitis remain elusive.

In this study, we first showed 1) that the expression of ET-1 was enhanced in IMQ-induced psoriasiform dermatitis as well as human psoriasis and 2) that the psoriasis-related cytokines IL-17 and TNF-α promoted ET-1 production from epidermal keratinocytes. As we previously reported that ET-1-primed DCs facilitate IL-17 production from T cells^18^, ET-1 from keratinocytes and IL-17 from T cells may form a feed-forward amplification network leading to psoriatic inflammation.

We next examined whether specific ET-1 receptor antagonists are able to inhibit psoriasiform dermatitis and interfere with the ET-1/IL-17 vicious cycle. The topical application of the ETAR selective antagonist ambrisentan significantly suppressed the clinical skin scores and the histological abnormalities such as epidermal hyperplasia and inflammatory cell infiltration, whereas the dual ETAR and ETBR antagonist bosentan exhibited limited inhibitory action on clinical scores and did not significantly inhibit the histological abnormalities. Although some differences in potency are observed between these two antagonists, similar trends towards reduced inflammation are clearly observed (Figs. 2–4). The observed variability may reflect differences in the bioavailability or potency of each antagonist, rather than distinct receptor biology. Previous studies have consistently demonstrated a significant involvement of ETAR but not ETBR in ET-1-induced cytokine production^31^ and expression of adhesion molecules^31^ as well as histamine release from mast cells^32^. Although keratinocytes express both ETAR and ETBR^30^, only the pretreatment with ETAR antagonist suppressed the ET-1-induced cytokine production from keratinocytes^30^. Besides, selective blockade of ETAR significantly reduced phenotypic and functional maturation of human DCs, whereas selective ETBR blockade showed conflicting effects^33^. Therefore, ETBR antagonist may exert contradictory effects against the ETAR signal in certain experimental conditions. Given that selective ETBR antagonist had no therapeutic effect (Fig. S1), our data also support this notion and that the ET-1 and ETAR axis plays, at least in part, an important role in the development of IMQ-induced psoriasiform dermatitis.

Since epidermal keratinocytes and infiltrated MHC class II-positive cells express ETAR (Fig. 5a), it was speculated that keratinocytes and DCs were the targets of ET-1 and ambrisentan. Especially for keratinocytes, because ET-1 has been demonstrated to promote their proliferation in an autocrine manner^34^, epidermal hyperplasia in psoriatic skin might be attributable to a self-regulatory effect of ET-1 on the proliferation of keratinocytes. In addition, ambrisentan decreased the expression of ET-1 in the epidermis (Fig. 3c). That is, ET-1 might promote its own production from keratinocytes in an autocrine manner, which is antagonized by ambrisentan. However, ET-1 had just a slight and not significant effect on the proliferation of NHEKs (Fig. S2). ET-1 stimulation did not induce ET-1 production from NHEKs (data not shown). These results suggested that, unexpectedly, the effect of ET-1 on epidermal keratinocytes was limited. In the evaluation of the effects of ET-1 and ambrisentan on another target, DCs, ambrisentan significantly inhibited the phenotypic and functional activation of sDCs in the regional LNs. These results reinforced the concept that the ET-1 and ETAR axis was operative in the activation of sDCs in IMQ-induced psoriasiform dermatitis. Thus, it seems more likely that ambrisentan attenuates psoriatic inflammation by antagonizing the effect of ET-1 on the interaction of DCs and T cells in LNs than by an effect on the proliferation of keratinocytes.

In summary, we have shown that the ET-1 and ETAR signalling pathway is an integral part of the pathogenesis of psoriasis. It is involved in the histological and molecular psoriatic signatures in IMQ-induced psoriasiform dermatitis. Keratinocytes and Th17 cells may also crosstalk with each other via ET-1 and IL-17A, respectively. The findings obtained here suggest that antagonizing the ETAR signal could be an alternative target in treating psoriasis.

## Methods

### Cell culture

Normal human epidermal keratinocytes (NHEKs) obtained from Lonza (Basel, Switzerland) were grown in culture dishes at 37 °C in 5% CO_2_^35^. The NHEKs were cultured in serum‐free keratinocyte growth medium supplemented with bovine pituitary extract, recombinant epidermal growth factor, insulin, hydrocortisone, transferrin, and epinephrine (all from Lonza). Culture medium was replaced every 2 days. Near confluence (70%–90%), cells were disaggregated with 0.25 mg/mL trypsin/0.01% ethylenediaminetetraacetic acid (Lonza) and subcultured. Second‐ to fourth‐passage NHEKs were used in all experiments. The cells (1 × 10^5^) were seeded in 24‐well culture plates, allowed to attach for 24 h, and then subsequently treated with or without IL‐17 or TNF-α (PeproTech, Rocky Hill, NJ, USA) for 24 h.

### ELISA

ET-1 in each culture supernatant was measured using cytokine-specific ELISA kits (BioSource and Endogen), in accordance with the manufacturers’ protocols.

### Mice

C57BL/6J mice (females, 6 to 8 weeks old) were obtained from CLEA Japan, Inc. The mice were maintained in a specific pathogen-free vivarium. All experiments were approved by the Animal Care and Experiment Committee of Kyushu University. All experimental methods were performed in accordance with the relevant guidelines and regulations.

### Development of psoriasiform dermatitis

Wild-type mice at 8 to 10 weeks of age received a daily topical dose of 62.5 mg of commercially available IMQ cream (5%, Beselna Cream; Mochida Pharmaceuticals, Tokyo, Japan) on the shaved back and both ears for 5 days, translating into a daily dose of 3.125 mg of the active compound. The control group was treated similarly with a vehicle cream (PROPETO®; Maruishi Pharmaceutical, Osaka, Japan). Ambrisentan (Wako, Osaka, Japan), bosentan (ChemScene, NJ, USA), or BQ-788 (Alomone labs, Jerusalem, Israel) was dissolved and diluted in ethanol (Nacalai Tesque, Kyoto, Japan) to achieve a final concentration of 5% and administered (20 µl on the ear and 180 µl on the back) from 4 days before IMQ application until the end of the study. As a control, vehicle (ethanol) was administered instead of ambrisentan, bosentan, or BQ-788.

### Scoring of disease activity

To evaluate the severity of inflammation of the back skin, an objective scoring system based on the clinical Psoriasis Area and Severity Index (PASI) was used, except that for the mouse model the affected skin area was not taken into account in the overall score^36^. Erythema, scales, and thickness were scored independently on a scale from 0 to 4: 0, none; 1, slight; 2, moderate; 3, marked; and 4, very marked. The cumulative score (erythema, scales, and thickness) served as a measure of the severity of inflammation (scale 0–12). The skin scores were measured by two investigators independently in a blinded manner.

### Histological examination and immunohistochemical staining

Samples from the ear and back after treatment with IMQ for five consecutive days were fixed in 10% formalin and then embedded in paraffin. Sections, 3 µm thick, were stained using H&E for evaluation of the numbers of infiltrating inflammatory cells and the thickness of the epidermis.

The sections were deparaffinized and rehydrated, and antigens were retrieved by Heat Processor Solution pH6 or Heat Processor Solution pH9 (Nichirei Biosciences Inc., Tokyo, Japan) at 100 °C for 40 min, followed by immersion in 3% H_2_O_2_ (Nichirei Biosciences Inc., Tokyo, Japan) for 10 min. Sections (mouse and human) for the staining of ET-1 were treated with Nichirei-Histofine® Mousestain kit (Nichirei Biosciences Inc.), in accordance with the instruction manual, followed by incubation with mouse monoclonal antibody against ET-1 (1:50; Abcam, Cambridge, UK) for 1 h at room temperature. The sections were then incubated with secondary antibody, Nichirei-Histofine® Simple Stain Mouse MAX-PO(M), for 30 min. Sections (mouse) for the staining of ETAR, IL-17A, and IL-23 were incubated with rabbit polyclonal antibody against ETAR (1:500; Novus, CO, USA), polyclonal antibody against IL-17A (1:100; Thermo Fisher, Tokyo, Japan), and polyclonal antibody against IL-23 (1:100; Abcam) at 4 °C overnight, followed by incubation with secondary antibody, Nichirei-Histofine® Simple Stain Mouse MAX-PO(R) (Nichirei Biosciences Inc.), for 60 min. Immunoreactivity was detected using 3,3-diaminobenzidine as a chromogen, followed by light counterstaining with haematoxylin. Sections stained without primary antibody served as a negative control. All formalin-fixed and paraffin-embedded tissues (samples from patients with psoriasis and from normal skin) were obtained from the archives of the Department of Dermatology, Kyushu University Hospital, Japan. All experimental methods were performed in accordance with the relevant guidelines and regulations. All experimental protocols were approved by Kyushu University and informed consent was obtained from all subjects.

### Quantitative reverse-transcription PCR (qRT-PCR) analysis

RNA was obtained from the ear skin by homogenizing with TRIzol® reagent (Thermo Fisher Scientific K.K., Tokyo, Japan). Total RNA was extracted from NHEKs using the RNeasy Mini kit and DNase protocol using RNase-Free DNase Set to remove contaminating genomic DNA (Qiagen, Hilden, Germany). Reverse transcription was performed using PrimeScript RT reagent kit (Takara Bio, Otsu, Japan). qRT-PCR was conducted on a CFX Connect Real-time System (Bio-Rad, Hercules, CA, USA) using SYBR Premix Ex Taq (Takara Bio). Amplification was initiated at 95 °C for 30 s as the first step, followed by 40 cycles of qRT-PCR at 95 °C for 5 s and at 60 °C for 20 s. mRNA expression was measured in quadruplicate and normalized to the β-actin expression level.

The sequences of primer pairs were as follows: forward primer for human EDN-1, 5′-AGAGTGTGTCTACTTCTGCCA-3′; reverse primer for human EDN-1, 5′-CTTCCAAGTCCATACGGAACAA-3′; forward primer for human β-actin, 5′-ATTGCCGACAGGATGCAGA-3′; reverse primer for human β-actin, 5′-GAGTACTTGCGCTCAGGAGGA-3′; forward primer for mouse IL-17a, 5′‐GGAGAGCTTCATCTGTGTCTCTG‐3′; reverse primer for mouse IL-17a, 5′‐TTGGCCTCAGTGTTTGGACA‐3′; forward primer for mouse TNF-α, 5′-GCCTCTTCTCATTCCTGCTTG-3′; reverse primer for mouse TNF-α, 5′-CTGATGAGAGGGAGGCCATT-3′; forward primer for mouse IL-12/IL-23 p40, 5′-GTTCGAATCCAGCGCAAGAA-3′; reverse primer for mouse IL-12/IL-23 p40, 5′-TTTGCATTGGACTTCGGTAGATGT-3′; forward primer for mouse IL-23 p19, 5′-AGCGGGACATATGAATCTACTAAGAGA-3′; reverse primer for mouse IL-23 p19, 5′-GTCCTAGTAGGGAGGTGTGAAGTTG-3′; and forward primer for mouse β-actin, 5′-GGCTGTATTCCCCTCCATCG-3′; reverse primer for mouse β-actin, 5′-CCAGTTGGTAACAATGCCATGT-3′.

### Antibodies and flow cytometry

The following fluorochrome-labelled anti-mouse monoclonal antibodies (mAbs) were obtained from BD Biosciences: CD11c (HL3), I-Ab (AF6-120.1), CD80 (16-10A1), and CD86 (GL1). Lymph node (LN) cells were washed in fluorescence-activated cell sorting (FACS) buffer (PBS/2% bovine serum albumin/0.1% azide), and 10^6^ cells/mL were incubated for 10 min at 4 °C with CD16/CD32 Fc block (BD Biosciences). Subsequently, cells were incubated for 30 min at 4 °C with the primary antibody or antibodies (1 mg/mL) and washed twice with FACS buffer^37^. To detect intracellular cytokine expression, cells were stimulated for 4 h in complete RPMI medium containing PMA (50 μg/ml), ionomycin (1 μg/ml), 10% FBS, 2 mM L-glutamine, 50 μM 2-mercaptoethanol, 1% penicillin-streptavidin, 1× monensin, and 1× Brefeldin A (BioLegend, San Diego, CA, USA). Cells were then fixed with 1% paraformaldehyde, permeabilized with 0.5% saponin, stained for intracellular cytokines, and analysed by flow cytometry. Samples were acquired on a FACS Canto (Becton Dickinson) and analysed with FlowJo (TreeStar).

### Cell proliferation assay

The proliferation of NHEKs was measured using WST-1 cell proliferation assay system (Takara Bio, Shiga, Japan), in accordance with the manufacturer’s protocol.

### Statistics

Statistical analysis was performed using GraphPad Prism Version 5 (GraphPad Software). Mann–Whitney U test, two-way ANOVA, and Student’s t-test were performed to determine the statistical significance of differences. A P value < 0.05 was considered significant.

## Supplementary information

Supplementary information.

## Data Availability

All data generated or analysed during this study are included in this published article and its Supplementary Information Files.
