# Supplementary material for: Topical application of endothelin receptor a antagonist attenuates imiquimod-induced psoriasiform skin inflammation
Source: Sci Rep. 2020 Jun 11;10:9510. doi: 10.1038/s41598-020-66490-z (PMC7289852; doi:10.1038/s41598-020-66490-z)
Supplement: Supplementary file 1 — Supplementary information. [file 41598_2020_66490_MOESM1_ESM.docx]

**Topical application of endothelin receptor A antagonist attenuates imiquimod-induced psoriasiform skin inflammation**

Takeshi Nakahara^1, 2, *^, Makiko Kido-Nakahara^2^, Dugarmaa Ulzii D^2^, Sho Miake^2^, Kei Fujishima^2^, Sawako Sakai^2^, Takahito Chiba^2, 3^, Gaku Tsuji^1, 4^, and Masutaka Furue^1, 2, 4^

^1^Division of Skin Surface Sensing, Graduate School of Medical Sciences, Kyushu University, Fukuoka, Japan

^2^Department of Dermatology, Graduate School of Medical Sciences, Kyushu University, Fukuoka, Japan

^3^Department of Dermatology and Plastic Surgery, Akita University Graduate School of Medicine, Akita, Japan

^4^Research and Clinical Center for Yusho and Dioxin, Kyushu University, Fukuoka, Japan

*corresponding author


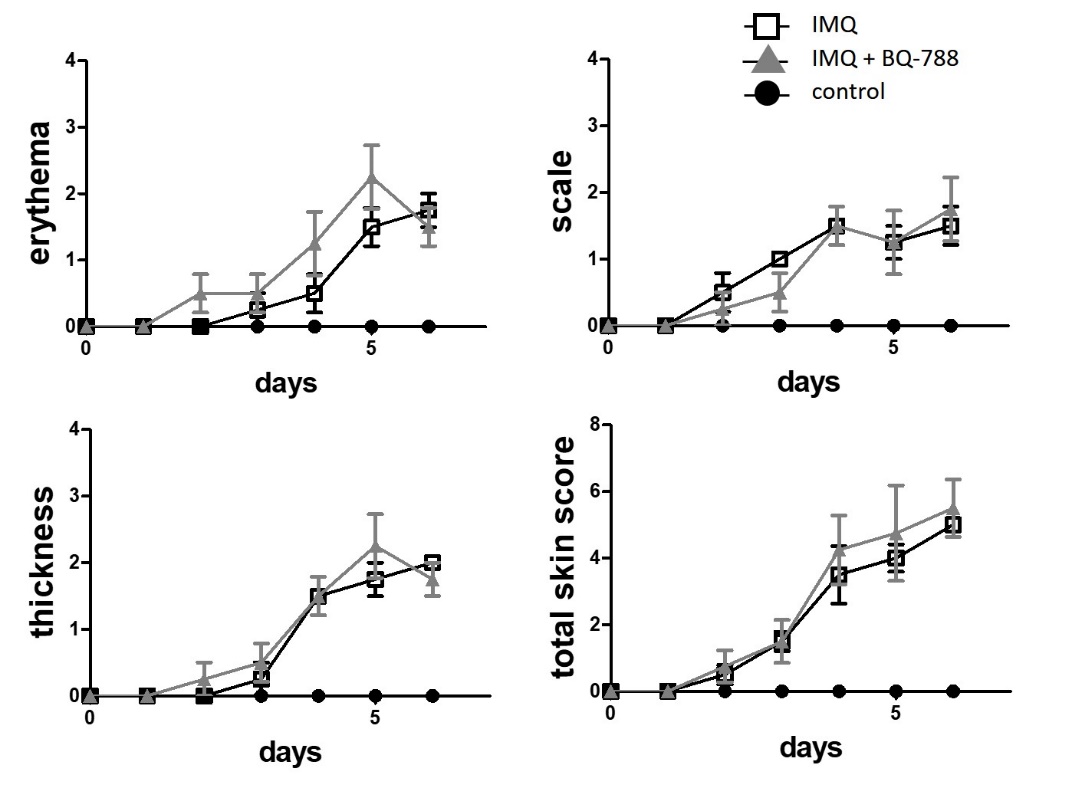


**Supplemental Fig. S1**

The effects of topical application of ETBR antagonist (BQ-788) on the clinical findings of IMQ-induced psoriasiform dermatitis. Shaved back skin and ears of B6 mice were topically treated with IMQ or control vehicle for six consecutive days. Topical BQ-788 was administered from 4 days before IMQ application until the end of the study. Clinical scores for disease severity were calculated daily using a scoring system based on the clinical Psoriasis Area and Severity Index. Erythema, scales, and thickness were scored independently on a scale from 0 to 4: 0, none; 1, slight; 2, moderate; 3, marked; and 4, very marked. The cumulative score (erythema, scales, and thickness) served as a measure of the severity of inflammation (scale 0–12). Results are representative of similar results obtained in two independent experiments. Data are presented as mean ± SEM (n=5 for each group).


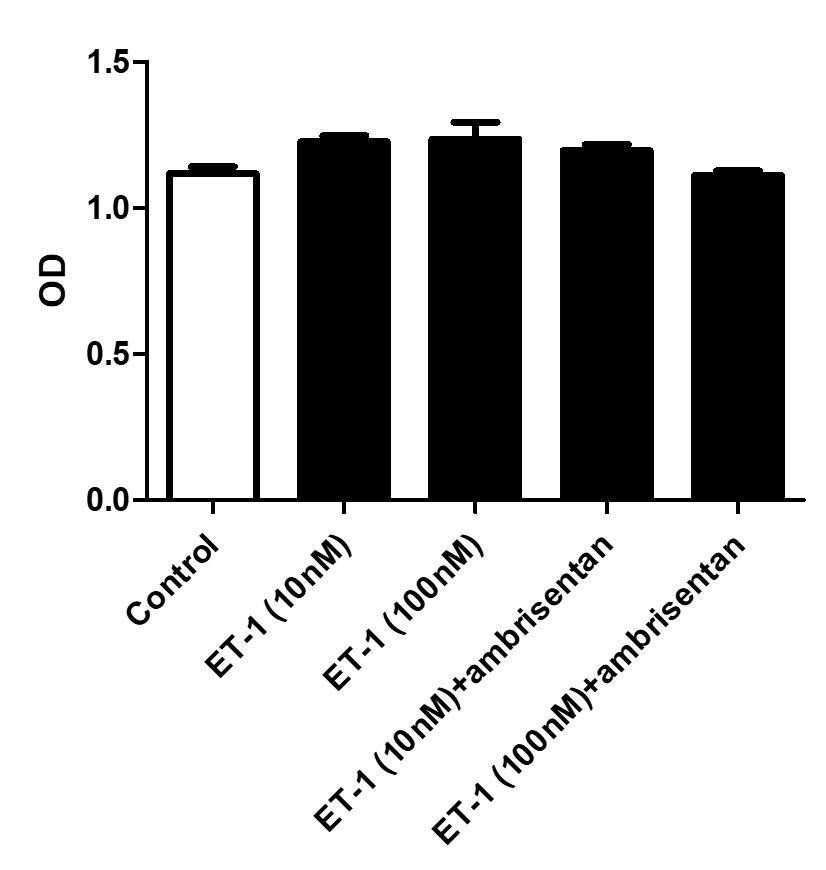


**Supplemental Fig. S2**

NHEKs were cultured with ET-1 in the presence or absence of ambrisentan for 24 h. Cell proliferation was determined using WST-1 assay. Data are presented as mean ± SEM. Results are representative of similar results obtained in two independent experiments.

**
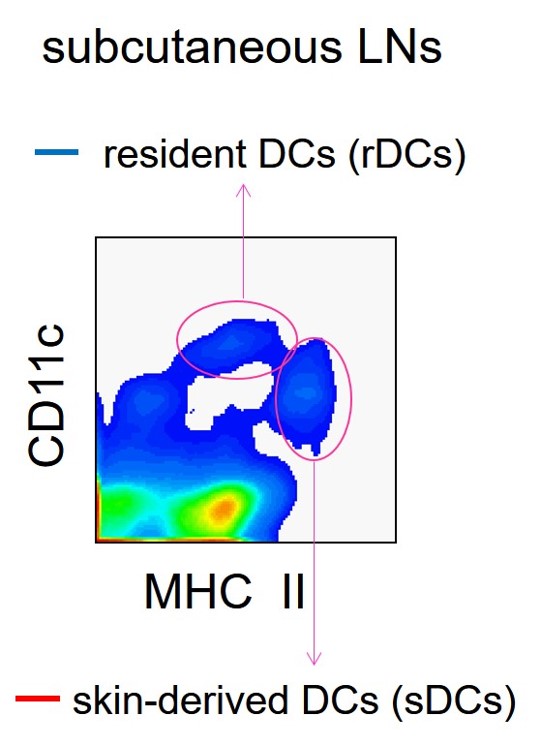
**

**Supplemental Fig. S3**

Different subsets of DCs in LNs stained for CD11C and MHC class II. Representative plots are shown.

**
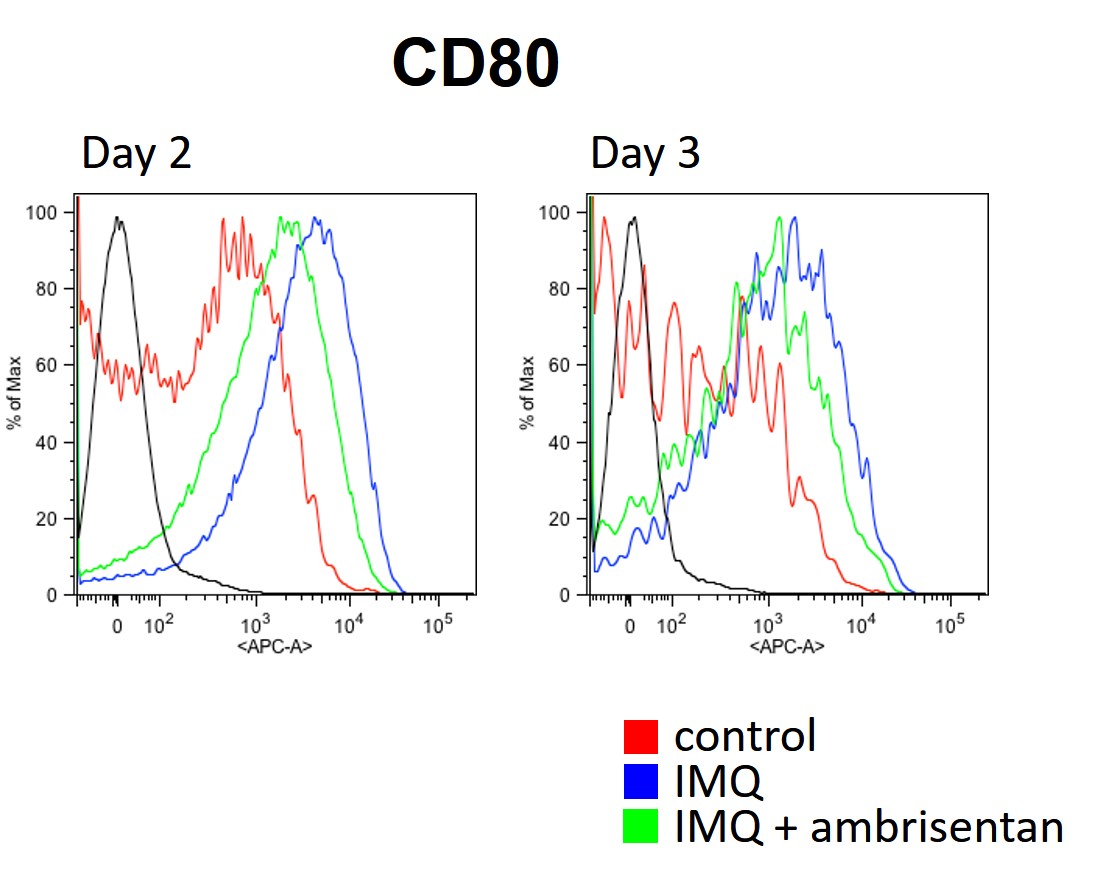
**

**Supplemental Fig. S4**

The expression of CD80 on skin-derived DCs in LNs after ambrisentan treatment. Inguinal LNs of control mice, IMQ-treated mice, and IMQ + ambrisentan-treated mice were harvested at day 2 or day 3. Results are representative of similar results obtained in two independent experiments.
